# Parental Resilience and Physical Health in Parents of Children With Type 1 Diabetes in Northern Greece

**DOI:** 10.7759/cureus.35149

**Published:** 2023-02-18

**Authors:** Aikaterini Chatzinikolaou, Kyriakos Kazakos, Dimitra-Anna Owens, Assimina Galli-Tsinopoulou, Aggeliki Kleisarchaki, Maria Lavdaniti

**Affiliations:** 1 Diabetes Mellitus Care, Department of Nursing, International Hellenic University, Thessaloniki, GRC; 2 Medical School, National and Kapodistrian University of Athens, Athens, GRC; 3 2nd Department of Pediatrics, Department of Medicine, AHEPA University General Hospital, Aristotle University of Thessaloniki, Thessaloniki, GRC

**Keywords:** parent education, parental wellbeing, children, parents, physical health, resilience, type 1 diabetes mellitus

## Abstract

Background: Type 1 diabetes mellitus (T1DM) is the most common endocrine and metabolic disorder in children. On the other hand, little is known regarding the health of parents whose children suffer from T1DM.

Aim: The study aims to investigate the mental resilience and physical health of parents of children with type 1 diabetes.

Methods: The sample consisted of 80 parents of children and adolescents with T1DM.The study was conducted with the contribution of associations of parents of children with type 1 diabetes in a large hospital in Northern Greece between April 2021 and September 2021. A demographic and clinical questionnaire, the Wagnild and Young Resilience Scale-14 (RS-14), and the General Health 28 Physical Health Measurement Questionnaire (GHQ-28) were used to collect the research data.

Results: Of the parents, 18.8% were male while 65% were female. The mean age of the parents was 44.02±6.71 years while the age of their children with diabetes was 13.13±6.05 years. Almost half of the children followed intensive insulin treatment (47.5%) whereas 22,5% reported that their children received insulin via a pump. A higher percentage of parents reported measuring their children's blood sugar more than six times a day (46,3%) and having their glycated hemoglobin (HbA1c) levels checked four times a year (51.2%). Finally, statistically significant effects on the physical symptoms and severe depression of parents of children with type 1 diabetes were observed.

Conclusions: Additional research is needed to assess the Greek parent population’s resilience and physical health. This study will help healthcare providers to expand their knowledge and meet parents’ needs.

## Introduction

Type 1 diabetes mellitus (T1DM) is one of the most common chronic diseases among children. Globally, it is estimated that 1.2 million children and adolescents suffered from T1DM in 2021 [1]. In Greece, the incidence of T1DM is estimated at 9.7 per 100,000 per year [2].

Resilience is defined as the ability to maintain physical and psychological well-being during an individual’s encounter with significant adversity or stressful situations [3]. Specifically, resilient individuals tend to exhibit adaptive behaviors, especially in areas such as social functioning and mental and physical health, and to experience positive emotions even amid stressful situations [4]. T1DM affects not only the children who suffer from it but also the entire family. Parents tend to have more stress and worry when caring for a child with T1DM, resulting in more family conflict and less social interaction [5].

Parents of children with T1DM are responsible for the majority of their child's T1DM management, a complex and time-consuming task that requires adherence to a T1DM care regimen involving frequent blood glucose monitoring, insulin administration, regulation of diet, and physical activity [6]. From this, we understand that the daily life of parents with children with T1DM can become demanding, leading to stress and anxiety.

Two recent studies have examined the impact of resilience and stress on the lives of parents with children suffering from T1DM. The results of the first survey showed that parents who had high resilience faced fewer depressive symptoms and their children had better glycemic control [7]. Additionally, the results of the second study showed that parents who reported higher resilience had a better quality of life and better mental health [5].

Recent studies also showed that parents’ emotional and physical adjustment can affect their children’s adherence and self-regulation. Furthermore, parental emotional burdens, including feelings of social isolation, fear of diabetes complications, and misunderstandings between children, can contribute to increased anxiety and depression among parents of children with T1DM [8-9]. Additionally, a systematic mixed-studies review, combining quantitative and qualitative research, examined the psychological experience of parents of children with T1DM. The study again showed the psychological distress of the parents [10].

Undoubtedly, there is a growing interest generally in the mental resilience and physical health of parents with children who have T1DM. Our research showed that few studies have been conducted internationally using different types of questionnaires concerning the resilience of these parents. To the best of our knowledge, no research using the Resilience Scale (RS-14) of Wanglid and Young and the General Health Questionnaire (GHQ-28) has been conducted in Greece. The purpose of this study was to assess the resilience and physical health of parents with children with T1DM in Greece using these two different instruments. Specifically, we aimed to assess the following research questions: (i) Is there a difference in parental demographics regarding parental resilience? (ii) Is there a difference in physical health depending on the parental demographics? (iii) Is there a difference in the mental resilience of the parents depending on the child's clinical characteristics? (iv) Is there any difference depending on the clinical characteristics of the child and the physical health of the parents? (v) What are the predicted factors that influence the mental resilience of parents? (vi) What are the predictive factors that affect the physical health of parents?

## Materials and methods

Study design and sample

This study was a cross-sectional study conducted in a convenience sample of 80 parents of children with T1DM in AHEPA University General Hospital, Thessaloniki, in Northern Greece between April 2021 and September 2021. The inclusion criteria for participants were that they were adult parents of children and adolescents with T1DM and had the ability to speak, read and write in Greek. All eligible participants provided written, informed consent before completing a structured questionnaire. Patients and treatment characteristics were collected from patients’ records. The study followed the declaration of Helsinki. The study was approved by the International Hellenic University Ethics Committee (approval number: 7/17-3-2021).

Instruments

The questionnaire consisted of three parts. The first part included the demographic characteristics of parents and clinical characteristics of children with T1DM. The second part measured the mental resilience of parents using the RS-14 by Wanglid and Young, and the third part concerned the physical health of parents using the GHQ-28.

The RS-14 of Wanglid and Young

The RS-14 is a seven-point Likert-type scale, with scores ranging from 1 (strongly disagree) to 7 (strongly agree). The sum of the score ranges from 14 to 98, with higher scores indicating stronger resilience. Scores above 90 indicate high resilience, while scores below 56 indicate very low resilience [4,11]. The RS-14 was translated into Greek by the Resilience Center (Montana, United States). The reliability of the questionnaire has been established in previous studies [11-14].

GHQ-28

The GHQ-28 was developed by Goldberg in 1978 and has since been translated into 38 languages. Developed as a screening tool to identify those likely to have or at risk of developing psychiatric disorders, the GHQ-28 is a 28-item measure of emotional distress in medical settings. Through factor analysis, the GHQ-28 has been divided into four subscales: physical symptoms, anxiety/insomnia, social dysfunction, and major depression.

There are different methods to score the GHQ-28. It can be scored from 0 to 3 for each response with a total possible score ranging from 0 to 84. Using this method, a total score of 23/24 is the threshold for the presence of distress. The bigger the score, above 24, the higher the distress. Alternatively, the GHQ-28 can be scored with a binary method where Not at all, and No more than usual score 0, and Rather more than usual and Much more than usual score 1 [15]. The number of symptoms is calculated across the GHQ-28 [16]. The validity of the GHQ-28 has been tested in various clinical settings and in a large number of cultures and languages. The GHQ-28 was translated into the Greek language by a group consisting of three psychiatrists and a clinical psychologist [17]. The reliability of the questionnaire has been established in previous studies [17-20].

Data analysis

Qualitative variables are described as n (%) whereas continuous variables are presented as mean ± standard deviation or as median (interquartile range). The normality of distribution was assessed using the Lillifors test. The association between the continuous variables was assessed by estimating the Spearman correlation coefficients. The effects of the demographic and clinical characteristics on the RS-14 score (normally distributed) were assessed using the independent t-test (for two groups) or the one-way ANOVA (for more than two groups). 

To allow analysis, quantitative variables (child’s age, age of diabetes diagnosis, and age of parent) were divided into two separate groups based on the median. The effects of the demographic and clinical characteristics on the General Health Questionnaire were assessed using the Mann-Whitney U test (for two groups) or the Kruskal Wallis H test (for three groups or more). On the Kruskal-Wallis test, significant main effects were followed by posthoc multiple comparisons with Dunn-Bonferonni Correction. For the statistically significant differences, Cohen’s d was computed using the platform Psychometrica™NPC (George, South Africa) [21].

Finally, a series of multiple regression analyses were conducted for each GHQ-28 subscale score to assess the predictors of compromised mental health. The first model (main analysis) included the RS-14 score as a predictor to assess whether resilience predicted scores on GHQ-28 whereas the second model (exploratory) added the demographic characteristics as predictors to assess whether there were additional variables explaining the total variance of the GHQ-28 scores. Data were analyzed using IBM SPSS Statistics for Windows, Version 25.0 (Released 2017; IBM Corp., Armonk, New York, United States). The significance level was set to p < 0.05.

## Results

Demographic, clinical, and psychometric characteristics

The mean age of the parents was 44.02±6.71 years, while the mean age of their children with T1DM was 13.13 ± 6.05 years old. The mean age of T1DM onset was 8.19±4.60 years. The children’s mean blood sugar level was 139.35 ± 55.52 mg/dL whereas mean glycated hemoglobin (HbA1c) level was 7.23 ± 1.70%. Almost half of the children followed intensive insulin treatment (47.5%) whereas 22,5% reported that their children received insulin via a pump. A higher percentage of parents reported measuring their children's blood sugar more than six times a day (46,3%) and having their HbA1c levels checked four times a year (51.2%). The demographic characteristics of parents and their children with diabetes are shown in Table 1.

**Table 1:** Demographic characteristics and diabetes-specific data of the participant and their children with T1DM Note: * n = 80; ^a^ With missing values T1DM: type 1 diabetes mellitus

Characteristics		n	%
Gender	Male	15	18.8
	Female	65	81.3
Age of participant^a^	<44 years old	38	48.7
	≥44 years old	40	51.3
Number of children	One child	18	22.5
	Two children	38	47.5
	Three children	20	25.0
	Four children	2	2.5
	> four children	2	2.5
Living Status	Lives with spouse and child/ren	68	85.0
	Lives only with their child/ren	12	15.0
Residence	Urban	51	63.7
	Semi-urban	21	26.3
	Rural	8	10.0
Mother’s employment status	unemployed	10	12.5
	homemaker	11	13.8
	Public servant	30	37.5
	Private sector worker	15	18.8
	farmer	1	1.3
	freelancer	10	12.5
	retired	3	3.8
Father’s employment status	unemployed	2	2.5
	Public servant	25	31.3
	Private sector worker	19	23.8
	farmer	5	6.3
	freelancer	25	31.3
	retired	4	5.0
Mother’s educational status	Primary School	2	2.5
	Middle School	2	2.5
	High school diploma	26	32.5
	Bachelor’s degree	34	42.5
	Postgraduate degree	14	17.5
	PhD	2	2.5
Father’s educational status	Primary School	3	3.8
	Middle School	2	2.5
	High school diploma	33	41.3
	Bachelor’s degree	28	35.0
	Postgraduate degree	7	8.8
	PhD	7	8.8
Children with diabetes gender	male	34	42.5
	female	46	57.5
Child’s with diabetes age	≤12 years old	42	52.5
	>12 years old	38	47.5
Age of diabetes diagnosis	< 8 years old	40	50.0
	≥ 8 years old	40	50.0
Insulin Therapy type	Conventional	24	30.0
	Intensive	38	47.5
	Pump	18	22.5
Blood sugar measurement frequency	1-2 times a day	10	12.5
	2-4 times a day	8	10.0
	4-6 times a day	25	31.3
	>6 times a day	37	46.3
HbA1c measurement frequency	1 time a year	3	3.8
	2 times a year	9	11.3
	3 times a year	27	33.8
	4 times a year	41	51.2
Blood sugar levels^a^	≤100 mg/dL	20	25.3
	≥100 mg/dL	59	74.7
HbA1c levels^a^	<7	47	60.3
	> 7	31	39.7

In Table 2, the descriptive characteristics of the GHQ-28 and the RS-14 are shown. Regarding internal consistency, all Cronbach’s alpha are satisfactory for both questionnaires.

**Table 2:** GHQ and RS descriptive characteristics GHQ: General Health Questionnaire; RS: Resilience Scale

	Minimum	Maximum	Mean	SD	Cronbach’s alpha
RS total	26.00	98.00	79.23	14.84	0.95
GHQ somatic	0.00	20.00	6.8	4.55	0.88
GHQ anxiety & insomnia	0.00	21.00	8.32	5.00	0.88
GHQ Social dysfunction	1.00	21.00	6.64	3.38	0.81
GHQ Severe depression	0.00	21.00	2.81	4.24	0.93
GHQ total scale	4.00	81.00	24.44	14.73	0.95
GHQ number of symptoms	0	28	5.94	6.55	-

Table 3 shows that almost half of the participants had more than four symptoms on the GHQ-28 (45.6%) while the 15.2% of parents reported having a low or very low resilience. 

**Table 3:** GHQ symptoms and RS categories based on score Note: ^a^ Missing values GHQ: General Health Questionnaire; RS: Resilience scale

GHQ number of symptoms categories^a^	n	%
<5 under cut-off (subclinical)	43	54.4
≥5 over cut-off (possibly clinical)	36	45.6
RS categories^a^	n	%
Very low (14-56)	6	7.6
Low (57-64)	6	7.6
On the low end (67-73)	8	10.1
Moderate (74-81)	24	30.4
Moderately High (82-90)	13	16.5
High (91-98)	22	27.8

Effects of demographic and clinical characteristics on resilience and mental health scores

Regarding resilience, parents that reported measuring blood glucose levels more than four times a day had a significantly lower RS-14 score (77.20±14.21) than those reporting measuring up to four times a day (86.1±15.26, t(77) = -2,30, p < 0.05, d = 0.62). No other effects on RS-14 scores were reported. Regarding mental health, no significant effects of participants’ gender, family status, living status, HbA1c measurement frequency, children’s blood sugar levels, and children’s insulin therapy type were noticed (p = ns).

Table 4 shows the means and standard deviations of GHQ-28 scores across the demographic and clinical characteristics in which significant effects on the scores were noticed. Significant main effects of the number of children were noticed on the total score (p = 0.02), on the GHQ-28 somatic score (p < 0.001), and on the GHQ-28 severe depression score (p = 0.04). Participants with two children had a higher median total score on the GHQ-28 (p = 0.013) and on the GHQ-28 somatic score (p = 0.03) than those with three children. In regards to the place of residence, participants had a higher median score on the total GHQ-28 scale (p = 0.03, d = 0.50) and in the number of symptoms (p = 0.03, d = 0.50). Regarding age, participants at least 44 years old had significantly lower score in the GHQ anxiety and insomnia subscale than the younger participants (p = 0.01, d = 0.63). Moreover, participants that had a child aged less than 12 years old had a significantly higher score in the GHQ-28 anxiety and insomnia subscale than those with older children (p = 0.01, d = 0.63). Moreover, participants that reported that their child with T1DM had HbA1c levels of more than 7% at the last measurement had a significantly lower median number of symptoms (p = 0.03, d = 0.52) and significantly lower score on anxiety and insomnia (p = 0.03, d = 0.51). Finally, participants that reported checking their blood sugar levels more than four times a day had a higher GHQ-28 social dysfunction score than those reporting measuring blood sugar levels up to four times a day (p = 0.02, d = 0.55).

**Table 4:** Significant effects of demographic and clinical characteristics of parents on the GHQ scores H: Kruskal-Wallis test; U: Mann-Whitney test; GHQ: General Health Questionnaire Superscripted letters refer to statistically significant difference between the respective groups after Dunn-Bonferroni

Variable		Median (IQR)	U or H (df)	p-value	Cohen’s d
GHQ total scale					
Number of children	a. One child	25 (17.50)	8.22 (2)	0.02	0.60
	b. Two children	26 (20.25)^c^			
	c. Three children	17 (11.50)^b^			
Place of residence	rural	25 (21)	514.5	0.03	0.50
	Semi-urban or rural	17 (14)			
GHQ number of symptoms					
HbA1c levels	<7%	4 (9.50)	501	0.03	0.52
	>7%	2 (7)			
Place of residence	rural	5 (10.25)	515	0.03	0.50
	Semi-urban or rural	2 (7)			
GHQ somatic					
Number of Children	a. One child	7 (5)	10.83	<0.001	0.73
	b. Two children	8 (7.50)^c^			
	c. Three children	3.50 (4.75)^b^			
GHQ anxiety & insomnia					
Age of the parent	<44 years old	11 (8)	483	0.01	0.63
	≥44 years old	6.50 (5.75)			
Age of the child	≤12 years old	8 (8)	544.00	0.02	0.54
	>12 years old	7 (6.50)			
HbA1c levels	<7%	8 (8)	506	0.03	0.51
	>7%	6 (8)			
GHQ Social dysfunction					
Blood sugar measurement frequency	1-4 times a day	4 (4.25)	342.50	0.02	0.55
	More than 4 times a day	7 (3)			
GHQ Severe depression					
Number of children	a. One child	1 (2.50)	6.29 (2)	0.04	0.49
	b. Two children	2 (3.25)			
	c. Three children	9 (3)			

Predictors of resilience of parents with children with diabetes

A regression model with demographic and clinical characteristics as predictors and RS-14 score as outcome was conducted. The model did not significantly explain the variance of the RS-14 score, R2 = 0.07, F(17, 75) = 1.33, p = 0.21. No predictors predicted the resilience score.

Predictors of the mental health of the parents with children with T1DM

For each subscale and total score of the GHQ-28, two separate regression models were conducted. The first model included the score on the RS-14 as a predictor which was followed by a second model (Enter Method) which included the demographic and clinical characteristics of the participants as predictors. The frequency of checking the HbA1c levels and age of the child were excluded from the model due to having high VIF levels.

Table 5 shows the predictors of the GHQ-28 total score and GHQ-28 number of symptoms. The first model on the total GHQ-28 score accounted for 26.4% of the total variance (F(1¸73) = 25.80, p < 0.001). The RS-14 score was a significant negative predictor of the GHQ-28 total score (b = -0.51). After including the demographic and clinical characteristics (second model), the model accounted for 51.1% of the total variance (F(16, 73) = 3.72, p < 0.001). The RS-14 score continued to be a significant negative predictor (b = -0.46). Living in an urban place (b = 0.28) and having two children (b = 0.39) were positive predictors of the total score. Reporting that their children receive intensive insulin treatment was a negative predictor of the total score (b= - 0.26).

**Table 5:** Multiple linear regressions on GHQ total score and GHQ symptoms Outcome: GHQ total score, R2 (model 1) = 0.26 (p < 0.001),  R2 (model 2) = 0.51 (p < 0.001)*p < 0.05, **p < 0.01, ***p < 0.001, α= reference group Outcome: GHQ symptoms, R2 (model 1) = 0.15 (p < 0.001),  R2 (model 2) = 0.47 (p < 0.01)*p < 0.05, **p < 0.01, ***p < 0.001, α= reference group GHQ: General Health Questionnaire; RS: Resilience scale

		GHQ total score					GHQ symptom				
Model	Predictors	B	SE B	β	95% CI		B	SE B	β	95% CI	
					LL	UL				LL	UL
1	(Constant)	63.89	7.96		12.24	27.97	20.10	3.94		12.24	27.97
	RS total score	-0.50	0.10	-0.51^***^	-0.28	-0.08	-0.18	0.05	-0.40^***^	-0.28	-0.08
2	(Constant)	72.01	17.16		6.10	39.23	22.66	8.27		6.10	39.23
	RS total score	-0.45	0.10	-0.46^***^	-0.25	-0.06	-0.16	0.05	-0.34^**^	-0.25	-0.06
	Age of participant	-0.31	0.22	-0.15	-0.34	0.08	-0.13	0.11	-0.14	-0.34	0.08
	Age of child’s diabetes diagnosis	-0.03	0.31	-0.01	-0.34	0.26	-0.04	0.15	-0.03	-0.34	0.26
	Urban Semi-urban^a^	7.98	3.08	0.28^*^	1.47	7.41	4.44	1.48	0.34^**^	1.47	7.41
	Blood sugar measurement frequency more than 4 times a day Blood sugar measurement frequency less than 4 times a day^a^	-0.28	3.83	-0.01	-4.00	3.39	-0.30	1.85	-0.02	-4.00	3.39
	Blood sugar levels ≥100 mg/dL Blood sugar levels <100 mg/dL^a^	0.04	3.15	0.00	-2.29	3.80	0.76	1.52	0.05	-2.29	3.80
	hba1c levels > 7 hba1c levels < 7^a^	-2.39	2.93	-0.09	-4.49	1.17	-1.66	1.41	-0.13	-4.49	1.17
	Male female^a^	-2.13	3.60	-0.06	-4.89	2.05	-1.42	1.73	-0.09	-4.89	2.05
	Lives with spouse^a^ Lives only with child	-0.80	4.15	-0.02	-4.75	3.27	-0.74	2.00	-0.04	-4.75	3.27
	Child male Child female^a^	-0.76	3.02	-0.03	-2.29	3.54	0.62	1.46	0.05	-2.29	3.54
	One child	6.35	3.97	0.20	-1.82	5.85	2.01	1.91	0.14	-1.82	5.85
	Two children Three or more children^a^	10.36	3.39	0.39^**^	1.38	7.92	4.65	1.63	0.38^**^	1.38	7.92
	Intensive insulin treatment	-6.92	3.34	-0.26^*^	-6.98	-0.54	-3.76	1.61	-0.30^*^	-6.98	-0.54
	Pump Conventional therapy^a^	-5.31	4.12	-0.17	-6.78	1.16	-2.81	1.98	-0.19	-6.78	1.16
	Mother employed Not employed^a^	-4.85	2.89	-0.17	-5.50	0.07	-2.71	1.39	-0.20	-5.50	0.07
	Father employed Not employed^a^	0.74	6.03	0.01	-4.63	7.01	1.19	2.91	0.05	-4.63	7.01

Regarding the number of symptoms, the first model on the total GHQ-28 score accounted for 15.9% of the total variance (F(1¸73) = 13.62, p < 0.001). The RS-14 score was a significant negative predictor of the GHQ-28 total score (b = -0.40). After including the demographic and clinical characteristics (second model), the model accounted for 32.3% of the total variance (F(16, 73) = 3.18, p = 0.001). The RS-14 score continued to be a significant negative predictor (b = -0.34). Living in an urban place (b = 0.34) and having two children (b = 0.38) were positive predictors of the total score. Reporting that their children receive intensive insulin treatment was a negative predictor of the total score (b= - 0.30).

Table 6 shows the predictors of the scores in the GHQ-28 somatic and anxiety and insomnia subscales. The first model on the somatic GHQ-28 score accounted for 13.6% of the total variance (F(1¸73) = 11.35, p = 0.001). The RS-14 score was a significant negative predictor of the GHQ-28 somatic score (b = -0.37). After including the demographic and clinical characteristics (second model), the model accounted for 45.5% of the total variance (F(16, 73) = 2.97, p = 0.001). The RS-14 score continued to be a significant negative predictor (b = -0.30). Having one child (b = 0.32) and having two children (b = 0.51) were positive predictors of the total score. Regarding the anxiety and insomnia subscale, the first model accounted for 16.3% of the total variance (F(1¸73) = 13.98, p < 0.001). The RS-14 score was a significant negative predictor of the GHQ-28 anxiety and insomnia score (b = -0.40). After including the demographic and clinical characteristics (second model), the model accounted for 43.9% of the total variance (F(16, 73) = 2.79, p = 0.002). The RS-14 score continued to be a significant negative predictor (b = -0.39). The age of the participant ((b = -0.31) was a negative predictor of the score whereas having one (b = 0.36) or two children (b = 0.40) were positive predictors.

**Table 6:** Multiple linear regressions on GHQ somatic and GHQ anxiety & insomnia Outcome: GHQ somatic, R2 (model 1) = 0.14 (p < 0.01),  R2 (model 2) = 0.46 (p < 0.01)*p < 0.05, **p < 0.01, ***p < 0.001, α= reference group Outcome: GHQ anxiety & insomnia, R2 (model 1) = 0.16 (p < 0.001),  R2 (model 2) = 0.44 (p < 0.01)*p < 0.05, **p < 0.01, ***p < 0.001, α= reference group GHQ: General Health Questionnaire; RS: Resilience scale

		GHQ somatic					GHQ anxiety & insomnia				
Model		B	SE B	β	95% CI		B	SE B	β	95% CI	
					LL	UL				LL	UL
1	(Constant)	16.18	2.87		10.47	21.89	19.35	3.03		13.31	25.40
	RS total score	-0.12	0.04	-0.37^**^	-0.19	-0.05	-0.14	0.04	-0.40^***^	-0.22	-0.07
2	(Constant)	16.01	6.03		3.95	28.08	32.85	6.57		19.70	45.99
	RS total score	-0.10	0.03	-0.30^**^	-0.17	-0.03	-0.14	0.04	-0.39^***^	-0.21	-0.06
	Age of participant	-0.09	0.08	-0.13	-0.24	0.07	-0.23	0.08	-0.32^**^	-0.40	-0.06
	Age of child’s diabetes diagnosis	0.05	0.11	0.05	-0.17	0.26	0.01	0.12	0.01	-0.22	0.25
	Urban Semi-urban^a^	2.16	1.08	0.23^*^	0.00	4.33	1.75	1.18	0.17	-0.61	4.11
	Blood sugar measurement frequency more than 4 times a day Blood sugar measurement frequency less than 4 times a day^a^	0.64	1.35	0.06	-2.05	3.34	-1.52	1.47	-0.13	-4.45	1.42
	Blood sugar levels ≥100 mg/dL Blood sugar levels <100 mg/dL^a^	-0.33	1.11	-0.03	-2.55	1.88	0.25	1.21	0.02	-2.17	2.66
	hba1c levels > 7 hba1c levels < 7^a^	-0.77	1.03	-0.08	-2.83	1.29	-1.67	1.12	-0.17	-3.92	0.57
	Male female^a^	-0.31	1.26	-0.03	-2.84	2.22	-0.91	1.38	-0.07	-3.66	1.85
	Lives with spouse^a^ Lives only with child	-0.11	1.46	-0.01	-3.03	2.81	0.01	1.59	0.00	-3.17	3.19
	Child male Child female^a^	-0.92	1.06	-0.10	-3.04	1.21	-1.89	1.16	-0.20	-4.20	0.43
	One child	3.38	1.39	0.32^*^	0.59	6.17	4.08	1.52	0.36^*^	1.04	7.12
	Two children Three or more children^a^	4.51	1.19	0.51^**^	2.13	6.89	3.78	1.30	0.40^*^	1.19	6.38
	Intensive insulin treatment	-2.29	1.17	-0.26	-4.63	0.06	-2.21	1.28	-0.23	-4.77	0.34
	Pump Conventional therapy^a^	-1.25	1.44	-0.12	-4.14	1.64	-2.17	1.57	-0.20	-5.32	0.98
	Mother employed Not employed^a^	-1.96	1.01	-0.20	-3.99	0.07	-1.07	1.11	-0.10	-3.28	1.14
	Father employed Not employed^a^	0.81	2.12	0.05	-3.43	5.04	-2.99	2.31	-0.16	-7.61	1.63

Table 7 shows the predictors of the scores in the GHQ-28 social dysfunction and Severe depression subscales. The first model on the social dysfunction GHQ-28 score accounted for 33.7% of the total variance (F(1¸73) = 36.64, p < 0.001). The RS-14 score was a significant negative predictor of the GHQ-28 social dysfunction score (b = -0.58). After including the demographic and clinical characteristics (second model), the model accounted for 44.4% of the total variance (F(16, 73) = 2.85, p = 0.002). The RS-14 score continued to be a significant negative predictor (b = -0.54). Living in an urban place was a positive predictor of the score (b = 0.24). Regarding the severe depression subscale, the first model accounted for 182% of the total variance (F(1¸73) = 15.99, p < 0.001). The RS-14 score was a significant negative predictor of the GHQ-28 severe depression score (b = -0.43). After including the demographic and clinical characteristics (second model), the model accounted for 23.8% of the total variance (F(16, 73) = 2.42, p = 0.007). The RS-14 score continued to be a significant negative predictor (b = -0.37). Living in an urban place was a positive predictor of the score (b = 0.33)

**Table 7:** Multiple linear regressions on GHQ social dysfunction and GHQ severe depression Outcome: GHQ Social dysfunction, R2 (model 1) = 0.34 (p < 0.01),  R2 (model 2) = 0.44 (p < 0.05)*p < 0.05, **p < 0.01, ***p < 0.001, α= reference group Outcome: GHQ Severe depression, R2 (model 1) = 0.18 (p < 0.001),  R2 (model 2) = 0.41 (p < 0.01)*p < 0.01, **p < 0.01, ***p < 0.001, α=reference group GHQ: General Health Questionnaire; RS: Resilience scale; Hba1c: Glycated hemoglobin

		GHQ social dysfunction					GHQ severe depression				
Model		B	SE B	β	95% CI		B	SE B	β	95% CI	
					LL	UL				LL	UL
1	(Constant)	16.46	1.66		13.15	19.77	11.90	2.35		7.22	16.58
	RS total score	-0.12	0.02	-0.58^***^	-0.17	-0.08	-0.12	0.03	-0.43^***^	-0.17	-0.06
2	(Constant)	13.95	4.02		5.90	21.99	9.21	5.30		-1.40	19.82
	RS total score	-0.12	0.02	-0.54^***^	-0.16	-0.07	-0.10	0.03	-0.37^**^	-0.16	-0.04
	Age of participant	0.01	0.05	0.02	-0.09	0.11	-0.01	0.07	-0.01	-0.14	0.13
	Age of child’s diabetes diagnosis	-0.03	0.07	-0.05	-0.18	0.11	-0.06	0.10	-0.07	-0.25	0.13
	Urban Semi-urban^a^	1.46	0.72	0.24^*^	0.02	2.90	2.61	0.95	0.33^***^	0.71	4.51
	Blood sugar measurement frequency more than 4 times a day Blood sugar measurement frequency less than 4 times a day^a^	0.26	0.90	0.04	-1.54	2.06	0.33	1.18	0.04	-2.03	2.70
	Blood sugar levels ≥100 mg/dL Blood sugar levels <100 mg/dL^a^	-0.27	0.74	-0.04	-1.75	1.21	0.40	0.97	0.05	-1.55	2.34
	Hba1c levels > 7 hba1c levels < 7^a^	-0.43	0.69	-0.07	-1.80	0.95	0.48	0.90	0.06	-1.33	2.29
	Male female^a^	-0.11	0.84	-0.01	-1.80	1.57	-0.81	1.11	-0.08	-3.03	1.42
	Lives with spouse^a^ Lives only with child	0.70	0.97	0.09	-1.24	2.65	-1.40	1.28	-0.13	-3.97	1.17
	Child male Child female^a^	0.46	0.71	0.08	-0.95	1.88	1.58	0.93	0.21	-0.28	3.45
	One child	-0.46	0.93	-0.07	-2.32	1.40	-0.64	1.23	-0.07	-3.10	1.81
	Two children Three or more children^a^	0.30	0.79	0.05	-1.29	1.88	1.78	1.05	0.24	-0.31	3.87
	Intensive insulin treatment	-0.60	0.78	-0.10	-2.16	0.97	-1.83	1.03	-0.24	-3.89	0.23
	Pump Conventional therapy^a^	-0.82	0.96	-0.12	-2.75	1.11	-1.07	1.27	-0.12	-3.61	1.48
	Mother employed Not employed^a^	-0.41	0.68	-0.07	-1.77	0.94	-1.41	0.89	-0.17	-3.20	0.37
	Father employed Not employed^a^	0.89	1.41	0.08	-1.94	3.71	2.04	1.86	0.14	-1.69	5.77

## Discussion

The objective of the study was to assess the mental resilience and physical health of parents with children who have T1DM. The study contributes to the growing body of evidence regarding the emotional and physical well-being of parents who have children suffering from T1DM and provides important information for parents, diabetes-specialized nurses, and school nurses as well. In this study, we found significant findings regarding resilience and physical health and how these contribute to social dysfunction, anxiety, and depression in parents of children with type 1 diabetes.

We are the first to use RS-14 and GHQ-28 scales to assess the resilience and physical health of parents with children who have T1DM in the Greek population. Other studies have mainly measured resilience using the Conor-Davidson Resilience Scale. 

In this study, we found that parents who reported measuring blood glucose levels more than four times a day had significantly lower resilience and greater social dysfunction than those reporting measuring up to four times a day. This finding is, to a certain extent, consistent with other studies, which showed that parents who tended to measure their children’s blood glucose four to six times per day demonstrated higher parental fear and concern for complications such as hypoglycaemic episodes [22,23].

A significant result of our study was the effect the number of children had on parents’ emotional and physical health. Particularly, parents with two children reported more physical symptoms and severe depression compared to parents with one or three children. To the best of our knowledge, this is an original finding. Further research is needed to draw safe conclusions.

Additionally, participants aged 44 or over had significantly lower scores in the GHQ-28 anxiety and insomnia subscale compared to younger participants in the survey. This finding is the first of its kind internationally and we assume that parents aged 44 or over have more experience and, as a result, lower stress levels because they know how to handle difficult situations that can arise from their child's T1DM, they can manage T1DM, and they can face the complications of the disease. Therefore, this finding of the research is justified due to the better control that experience has given to parents aged 44 or over, resulting in lower anxiety and better sleeping habits/lack of sleeping disorders.

Moreover, participants who had a child aged less than 12 years old had a significantly higher score in the GHQ-28 anxiety and insomnia subscale than those with older children. These results are consistent with previous research, which has demonstrated that parents of younger children with T1DM experience sleep disruption and anxiety [6,24,25].

Moreover, participants who reported that their child with T1DM had HbA1c levels of more than 7% at the last measurement had a significantly lower median number of symptoms and significantly lower scores on anxiety and insomnia. We assume that parents know how to manage T1DM, checking their children’s blood glucose frequently on a daily basis, and, as long as the blood measurements are within the permissible limits, there is no reason to be stressed. Additionally, the HbA1c examination takes place three to four times per year, so maybe parents believe that it can be fixed in the next HbA1c examination. However, this finding is inconsistent with the results of a recent study that showed that parents of children with higher HbA1c tend to have more anxiety and concern about the complications that can occur from T1DM [26].

Limitations

This study has some limitations. It was conducted in one hospital located in a major Greek city, with a relatively small sample of parents and simultaneously via an online form filled by associations of parents of children with T1DM, so the results cannot be generalized to the entire Greek population. Another limitation was the short timeline of the study, which was conducted over six months. However, the results provide valuable information on the issue at hand and illustrate the great need for further research to draw reliable conclusions. Despite these limitations, our study has one significant strength: to the best of our knowledge, this is the first population-based study to use the combination of RS-14 and GHQ-28 scales to investigate the mental resilience and physical health of parents of children with T1DM in Greece.

## Conclusions

Our study confirms that resilience and physical health play a significant role among parents of children with T1DM. Glucose monitoring, measurements of HbA1c, depression, anxiety, insomnia and age, according to our study, are associated with parents’ social well-being and their everyday life. This study adds to the growing interest in the psychosocial and physical well-being of parents of children with T1DM. Nevertheless, further research is needed to evaluate the mental resilience and physical health of Greek parents of children with T1DM. The results of this study will help parents to cope with difficult mental, emotional, and social dysfunctions, and healthcare professionals to expand their knowledge and meet parents’ needs.
